# Selective mitochondrial superoxide generation *in vivo* is cardioprotective through hormesis

**DOI:** 10.1016/j.freeradbiomed.2019.01.034

**Published:** 2019-04

**Authors:** Salvatore Antonucci, John F. Mulvey, Nils Burger, Moises Di Sante, Andrew R. Hall, Elizabeth C. Hinchy, Stuart T. Caldwell, Anja V. Gruszczyk, Soni Deshwal, Richard C. Hartley, Nina Kaludercic, Michael P. Murphy, Fabio Di Lisa, Thomas Krieg

**Affiliations:** a Department of Biomedical Sciences, University of Padova, 35131, Padova, Italy; b Department of Medicine, University of Cambridge, Hills Road, Cambridge, CB2 0XY, UK; c Mitochondrial Biology Unit, University of Cambridge, Hills Road, Cambridge, CB2 0XY, UK; d School of Chemistry, University of Glasgow, Glasgow, G12 8QQ, UK; e Neuroscience Institute, National Research Council of Italy (CNR), 35131, Padova, Italy

## Abstract

Reactive oxygen species (ROS) have an equivocal role in myocardial ischaemia reperfusion injury. Within the cardiomyocyte, mitochondria are both a major source and target of ROS. We evaluate the effects of a selective, dose-dependent increase in mitochondrial ROS levels on cardiac physiology using the mitochondria-targeted redox cycler MitoParaquat (MitoPQ). Low levels of ROS decrease the susceptibility of neonatal rat ventricular myocytes (NRVMs) to anoxia/reoxygenation injury and also cause profound protection in an *in vivo* mouse model of ischaemia/reperfusion. However higher doses of MitoPQ resulted in a progressive alteration of intracellular [Ca^2+^] homeostasis and mitochondrial function *in vitro*, leading to dysfunction and death at high doses. Our data show that a primary increase in mitochondrial ROS can alter cellular function, and support a hormetic model in which low levels of ROS are cardioprotective while higher levels of ROS are cardiotoxic.

## Teaser

Antonucci et al. demonstrate that a primary increase in superoxide selectively within the mitochondria directly impacts on mitochondrial and cell function. Moreover, a slight increase in mitochondrial ROS is protective against cardiac ischaemia-reperfusion injury despite negative effects at higher concentrations.

## 1 Introduction

Ischaemia/reperfusion (I/R) injury occurs when the blood supply to a region of tissue is disrupted and later restored. Key to the development of the pathology is a lack of oxygen for oxidative phosphorylation within mitochondria. This results in the arrest of forward electron flow in the respiratory chain [1] due to the lack of oxygen to act as the terminal electron acceptor. Consequently, succinate is accumulated during ischaemia by the reduction of fumarate at mitochondrial complex II [2], or from anaplerotic supply of glutamate to the tricarboxylic acid cycle leading to succinate that cannot be oxidized due to the reduced Coenzyme Q pool [3]. At reperfusion, the re-introduction of oxygen leads to sudden changes in myocardial viability, mediated by a burst of ROS production within the mitochondria. While a number of sources of ROS have been proposed to be responsible, recently it has been shown that the succinate pool accumulated during ischaemia is rapidly oxidized upon reperfusion, driving ROS production from complex I through reverse electron transport [2]. The sustained opening of the mitochondrial permeability transition pore (mPTP), a key arbiter of cell fate, also results in significant ROS production. As ROS are among the factors contributing to prolonged mPTP opening there is a positive feedback loop of ROS-induced ROS release [4] through which ROS generation results in sustained mitochondrial dysfunction and eventually cell death [5].

However, aside from their role in driving mPTP-mediated cell damage, mitochondrial ROS production also impacts positively on several aspects of myocardial I/R injury. Mitochondrial ROS play crucial roles in signalling processes both within mitochondria and in their interplay with other cellular sites [6]. Indeed, the protective mechanism underlying ischaemic preconditioning (IPC) appears to involve generation of low levels of ROS [[7], [8], [9]]. This has been demonstrated by showing that the protective effects of an IPC protocol may be abrogated by antioxidants [[10], [11], [12], [13], [14], [15], [16]], or replicated by the addition of exogenous oxidants in a pre- or post-conditioning like manner [[17], [18], [19]]. Indeed, it has been demonstrated that the acute treatment with H_2_O_2_ in a model of Langerdorff perfused hearts elicits cardioprotection in a preconditioning-like manner depending of its concentration [20,21]. These findings that ROS can be either cardiotoxic or cardioprotective, depending on the context, may help explain why the use of general ROS scavengers has yielded mixed results. For example there are also studies in experimental models that report no effect of antioxidants upon I/R injury [[22], [23], [24], [25]]. In the clinic, large scale trials with antioxidants have produced equivocal effects upon cardiovascular health outcomes [26,27]. These findings are also consistent with the growing appreciation for the differential effects of “good” and “bad” ROS [[28], [29], [30]], by which ROS can either contribute to cell death, or at low levels activate cardioprotective mechanisms: a concept referred to as ‘hormesis’.

The context in which ROS might lead to these opposing consequences will arise due to differential effects of ROS production in three primary dimensions: in magnitude, in timing, and in spatial distribution. A better understanding of, and control of these dimensions over ROS production may provide mechanistic insights as to how ROS can have different effects. Here, we address this point by exploring the role of ROS generated within mitochondria at different concentrations, and characterize the processes linking changes in mitochondrial ROS levels to cardiac pathophysiology with focus on cardiac I/R injury. To investigate the consequences of a primary increase in ROS specifically within the mitochondrial compartment, we used the mitochondria-targeted compound MitoParaquat (MitoPQ) [31]. MitoPQ was designed based upon the conjugation of a paraquat moiety (1,1’-dimethyl-4,4’-bipyridinium dichloride) [32] with the mitochondria targeting triphenylphosphonium group. At the flavin site of complex I in the electron transport chain, MitoPQ accepts an electron to generate a radical monocation that reacts rapidly with oxygen to specifically generate superoxide, which is the proximal ROS species produced endogenously [33].

In this work we demonstrate that a selective primary increase in mitochondrial ROS is a causal factor in changes of mitochondrial and cell function, and show that low levels of mitochondrial ROS are protective against ischaemic injury both *in vitro* and *in vivo*, while higher levels are damaging.

## 2 Results

### 2.1 MitoPQ induces a primary increase in mitochondrial ROS levels in a dose-dependent manner

To investigate the effects of mitochondrial ROS production by MitoPQ in cardiomyocytes, neonatal rat ventricular myocytes (NRVMs) were treated for 2 h with different doses of MitoPQ. High levels of ROS were assessed with a reduced form of MitoTracker Red (MTR) that fluoresces only following oxidation. However, MTR presents some limitations due to its relative lack of sensitivity and specificity, that its accumulation depends on cell and mitochondrial membrane potential (ΔΨm), and these can be affected by different treatments independently of ROS formation. Therefore, to detect low levels of ROS we used MitoHyPer, a genetically encoded sensor that is highly specific and sensitive for sub-micromolar concentrations of mitochondrial H_2_O_2_ [34].

**Fig. 1:**
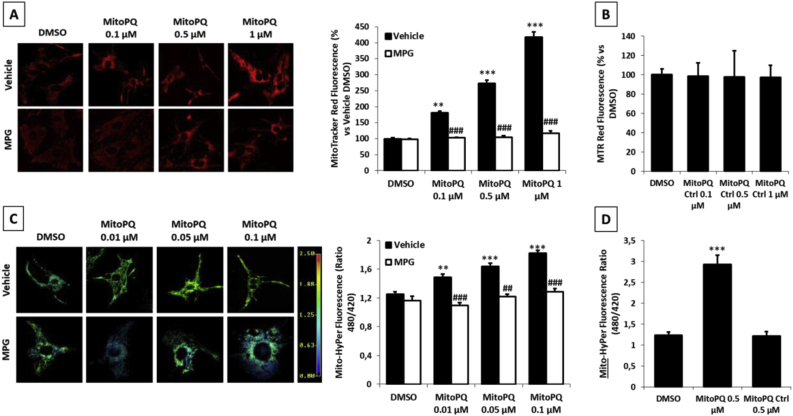
**Effect of MitoPQ on ROS formation.** Mitochondrial ROS formation monitored by: **A)** MTR in isolated NRVMs treated for 2 h with different concentrations of MitoPQ or **B)** MitoPQ Control Compound, with or without 30 min of pretreatment with 500 μM MPG. Scale Bar: 30 μm. **C)** Mitochondrial H_2_O_2_ formation measured by MitoHyPer in isolated NRVMs treated for 2 h with different concentrations of MitoPQ, with or without 30 min of pretreatment with 500 μM MPG. Scale Bar: 20 μm **D)** Mitochondrial H_2_O_2_ formation measured by MitoHyPer in isolated NRVMs treated for 2 h with 0.5 μM MitoPQ or 0.5 μM MitoPQ Control Compound. Approximately 70 cells were analysed per condition in each experiment and all the experiments were performed at least three times. Data are expressed as mean ± SEM. *p < 0.05, **p < 0.01, ***p < 0.001 vs DMSO vehicle; #p < 0.05, ##p < 0.01, ###p < 0.001 vs MitoPQ.

MitoPQ caused a significant increase in mitochondrial ROS levels in a dose-dependent manner that was not observed when cells were pre-treated with the antioxidant N-(2-Mercaptopropionyl)glycine (MPG) (Fig. 1A–C). Cells treated with a MitoPQ control compound (Supplementary Fig. 1) that is structurally similar to MitoPQ but which does not undergo redox cycling did not show an increase in mitochondrial ROS levels (Fig. 1B–D, Supplementary Fig. 2A and B). Moreover, cells treated with non-mitochondrial paraquat (PQ) did not show an increase in mitochondrial ROS levels (Supplementary Fig 2C), in line with previous reports [31].

Taken together, these data confirm that MitoPQ produces ROS within mitochondria in a dose-dependent manner.

### 2.2 A primary increase in mitochondrial ROS levels affects mitochondrial function in a dose-dependent manner

**Fig. 2:**
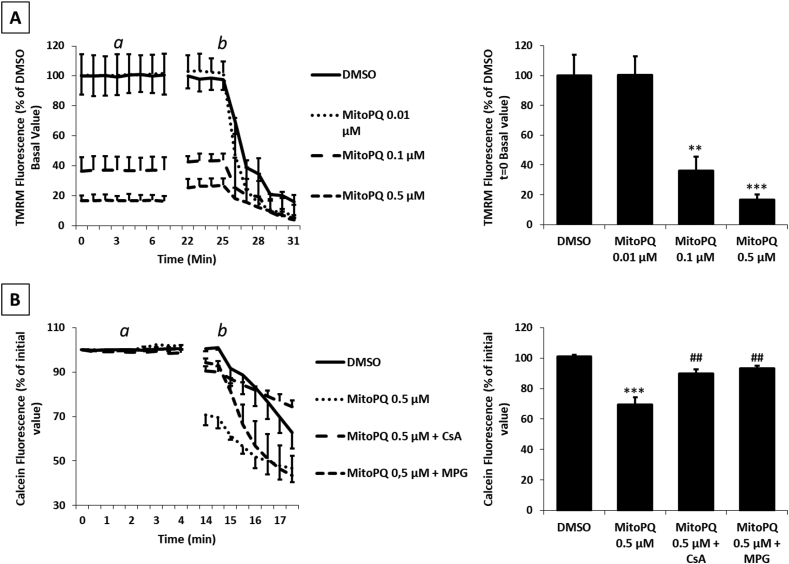
**Effects of MitoPQ on mitochondrial membrane potential and mPTP opening. A)** Mitochondrial membrane potential (ΔΨm) monitored by TMRM fluorescence in isolated NRVMs following incubation for 2 h with MitoPQ at different concentrations *a*: 4 μM oligomycin, *b*: 4 μM FCCP. **B)** mPTP opening monitored by decrease of calcein fluorescence in isolated NRVMs pretreated for 30 min with or without 1 μM CsA or 500 μM MPG. *a*: treatment with DMSO as control or 0.5 μM MitoPQ; *b*: 5 μM Calcimycin. Data were quantified 12 min after treatment with MitoPQ/DMSO. Approximately 30 cells were analysed per condition in each experiment and all the experiments were performed at least three times. Data are expressed as mean ± SEM. *p < 0.05, **p < 0.01, ***p < 0.001 vs DMSO vehicle; #p < 0.05, ##p < 0.01, ###p < 0.001 vs MitoPQ.

An increase in mitochondrial ROS production has been reported to induce a decrease in ΔΨm that is associated with mPTP opening [35,36]. To investigate whether MitoPQ-induced ROS affect mitochondrial function, we monitored ΔΨm using tetramethylrhodamine fluorescence. NRVMs treated with different doses of MitoPQ displayed a dose-dependent decrease in ΔΨm (Fig. 2A). Notably, although promoting H_2_O_2_ formation (Fig. 1C), 0.01 μM MitoPQ did not affect ΔΨm. However, a defective electron transport chain may not lead to a detectable decrease in ΔΨm, since in isolated cells the proton gradient can be maintained by the reverse activity of F_O_F_1_ ATP synthase [37]. This compensatory process was abolished by the presence of the F_O_F_1_ ATPase inhibitor oligomycin in all the experiments. The lack of ΔΨm variations at low MitoPQ doses suggests that mild ROS formation is unlikely to alter electron transport chain function. Taken together, these results show that a primary increase in mitochondrial ROS formation decreases ΔΨm in a dose-dependent manner, but that this does not occur at low ROS levels.

Analysing the causal relationship between mPTP, ROS formation and ΔΨm is complicated by the fact that mPTP opening is both a cause and a consequence of ΔΨm loss and mitochondrial ROS generation [[38], [39], [40]]. Hence, we used MitoPQ to investigate the effects of a primary increase in mitochondrial ROS levels on mPTP induction assessed by monitoring calcein fluorescence [41,42]. NRVMs were pre-treated with or without cyclosporine A (CsA), a desensitizer of the mPTP. Since CsA is also an inhibitor of the multidrug resistance P-glycoproteins (MDR), which may alter MitoPQ distribution, cells were pre-treated also with cyclosporine H which inhibits the MDRs but does not affect mPTP opening [43]. Treatment with 0.5 μM MitoPQ induced a rapid decrease in calcein fluorescence (∼30%) that was prevented by CsA. MitoPQ-induced mPTP opening was dependent on ROS formation since it was abrogated by MPG. Notably, MPG did not display any effect when mPTP opening was induced by calcimycin (Fig. 2B). Taken together, these results indicate that ROS induced by 0.5 μM MitoPQ lead to mPTP opening in NRVMs.

### 2.3 A primary increase in mitochondrial ROS levels affects cell function and viability

**Fig. 3:**
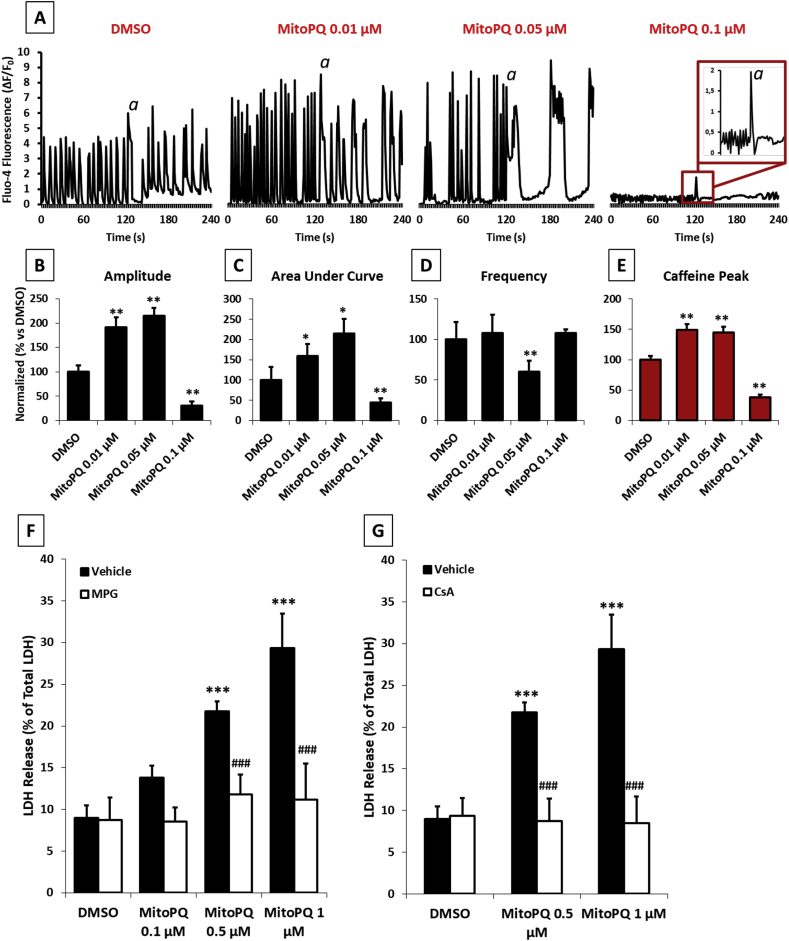
**Effects of MitoPQ on cytosolic [Ca**^**2+**^**] homeostasis and cell viability. A-E)** cytosolic [Ca^2+^] homeostasis monitored by Fluo-4 AM in isolated NRVMs treated for 2 h with different concentrations of MitoPQ. **A)** Comparison between cytosolic Ca^2+^ oscillatory patterns. *a*: 10 mM caffeine. **B)** Peak amplitude average; **C)** Area Under Curve (AUC) average; **D)** Peaks Frequency average; **E)** Average caffeine Peak amplitude. **F-G)** Cell death monitored by LDH released from isolated NRVMs treated for 24 h with different concentrations of MitoPQ and pretreated with or without **F)** 500 μM MPG or **G)** 1 μM CsA. *A-E:* Approximately 30 cells were analysed per condition in each experiment and all the experiments were performed at least three times. Data are expressed as mean ± SEM. *F-G:* Approximately 4 wells (cell density: 1.5 × 10^5^ cells/well in 24 wells plates) were analysed per condition in each experiment and all the experiments were performed at least three times. Data are expressed as mean ± SEM. *p < 0.05, **p < 0.01, ***p < 0.001 vs DMSO vehicle; #p < 0.05, ##p < 0.01, ###p < 0.001 vs MitoPQ.

We hypothesized that mitochondrial ROS would affect cellular sites and functions outside the mitochondria. In particular, our attention was focused on [Ca^2+^]_I_ homeostasis due to its central role in cardiac physiology [44,45]. Treatment with 0.01 μM MitoPQ caused a significant increase in both amplitude (Fig. 3B) and response of the sarcoplasmic reticulum to caffeine (Fig. 3E), without affecting the oscillatory pattern or the frequency of the Ca^2+^ transients (Fig. 3A–D). In contrast, doses of MitoPQ >0.01 μM induced dose-dependent alterations in all the oscillatory parameters. Notably, increasing MitoPQ concentration to 0.1 μM disrupted [Ca^2+^]_I_ homeostasis, causing the cells to become unexcitable (Fig. 3). Overall, these findings demonstrate that ROS produced within mitochondria alter [Ca^2+^]_I_ homeostasis within the cytosol and endoplasmic reticulum with different amounts of ROS causing different effects.

Since oxidative stress associated with mitochondrial dysfunction is known to decrease cell viability [46,47], we investigated whether MitoPQ-induced oxidative stress could lead to cell death in NRVMs. The loss of cell viability was measured as lactate dehydrogenase (LDH) release in NRVMs treated for 24 h with increasing doses of MitoPQ. MitoPQ doses ≥0.5 μM significantly increased cell death (∼30%) which could be abrogated by the addition of the free radical scavenger MPG (Fig. 3F). Notably, 0.1 μM MitoPQ did not significantly alter cell viability despite promoting ROS formation, mitochondrial dysfunction and [Ca^2+^]_I_ dyshomeostasis without mPTP opening. Therefore, we hypothesized that the decreased cell viability at high concentrations of MitoPQ was related to mPTP opening [48,49]. This was confirmed by CsA treatment which prevented cell death induced by MitoPQ at doses ≥0.5 μM (Fig. 3G).

### 2.4 Low levels of MitoPQ-induced ROS reduce cell death following anoxia/reoxygenation

**Fig. 4:**
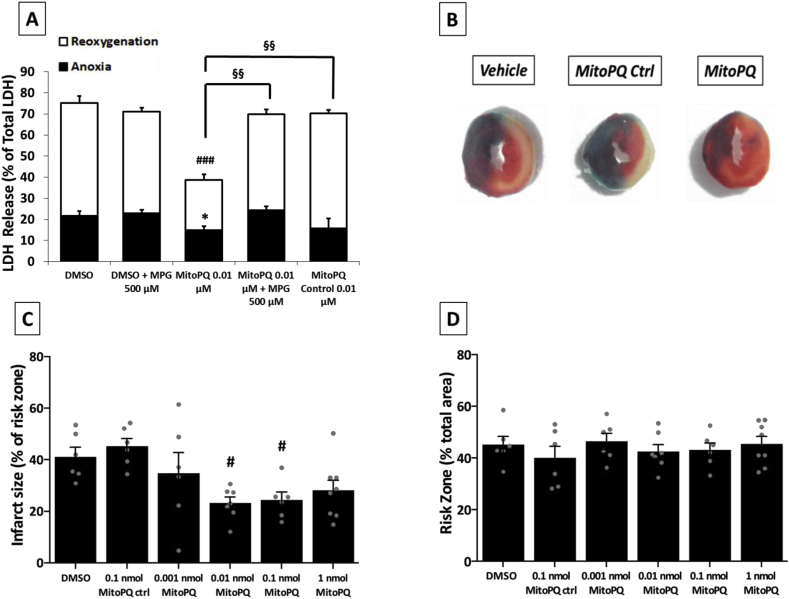
**Effects of MitoPQ on cardiomyocytes viability both *in vitro* and *in vivo*. A)** Cell death measured by LDH release from isolated NRVMs treated for 2 h with 0.01 μM MitoPQ or 0.01 μM MitoPQ control compound, with or without 500 μM MPG. *p < 0.05 vs DMSO Anoxia, ###p < 0.001 vs DMSO Reoxygenation, §§p < 0.01 vs MitoPQ. **B)** Representative infarct slices from hearts treated with vehicle, MitoPQ control compound 0.01 nmol MitoPQ. **C)** Infarct size in hearts of mice exposed to acute myocardial I/R, in presence or absence of 0.01 nmol MitoPQ control compound or the indicated dose of MitoPQ. *p < 0.05 vs DMSO. **D)** Risk zone in heart of mice exposed to acute myocardial I/R, in presence or absence of 0.01 nM MitoPQ control compound or the indicated dose of MitoPQ. *A:* at least 4 wells (cell density: 1.5 × 10^5^ cells/well in 24 wells plates) were analysed per condition in each experiment and all the experiments were performed at least three times. Data are expressed as mean ± SEM. *B-D: n = 6-8.* Data are expressed as mean ± SEM. #p < 0.05. Overall Anova p = 0.0053.

We demonstrated that low doses of MitoPQ (0.01 μM) elevated ROS but did not alter mitochondrial function, [Ca^2+^]_I_ homeostasis and cell viability. Since an increase in mitochondrial ROS levels have been proposed to be involved in IPC [5,[50], [51], [52]], we hypothesized that a primary increase in non-toxic mitochondrial ROS levels by MitoPQ might enhance tolerance to post-anoxic injury. To assess this, we evaluated whether the low dose of MitoPQ could mimic the protection elicited by IPC against anoxia/reoxygenation injury. Cells pre-treated with 0.01 μM MitoPQ, with or without the antioxidant MPG, were exposed to 12 h of anoxia followed by 1 h of reoxygenation. MitoPQ treatment significantly decreased cell death both at the end of anoxia and following reoxygenation. The cardioprotective effect of MitoPQ was lost in cells treated with MPG (Fig. 4A). In addition, an identical concentration of the MitoPQ control compound did not induce any protection. Notably, the reliability of the protocol has been assessed by the protective effect elicited by CsA in NRVMs exposed to anoxia/reoxygenation (Supplementary Fig. 2D). These data indicate that the increase in cell viability was due to MitoPQ-induced ROS.

### 2.5 MitoPQ reduces infarct size in an *in vivo* model of ischaemia/reperfusion injury

Finally, we utilised an *in vivo* surgical model of acute myocardial I/R injury in the mouse to determine the effect of a primary increase of ROS produced by MitoPQ upon infarct size. A bolus of MitoPQ was given by i.v. injection 15 min prior to the onset of cardiac ischaemia produced by occlusion of the left anterior descending coronary artery. This was followed by 2 h reperfusion. Elevating mitochondrial ROS by intermediate doses of MitoPQ (i.e. 0.01–0.1 nmol) significantly reduced infarct size compared to both DMSO-only control or MitoPQ control compound (Fig. 4B-C-D). However, at both lower or higher doses of MitoPQ no protection was observed, and a dose of 5 nmol MitoPQ was fatal. This shows that low levels of mitochondrial ROS can be cardioprotective during I/R injury *in vivo*, while higher levels of ROS cause damage.

Considering the changes caused in cellular calcium dynamics observed following treatment of NRVMs with MitoPQ, we evaluated its effects on haemodynamics *in vivo* using dynamic measurements of left ventricular pressure and volume. However no significant difference was observed between animals injected with the most cardioprotective dose of MitoPQ compared with hearts treated with vehicle only (Supplementary Table 1).

## 3 Discussion

This study demonstrates that the primary formation of mitochondrial ROS exerts differential effects upon the heart during acute myocardial I/R injury, with cardioprotection conferred by a narrow intermediate dose range whilst no reduction in infarct size was observed at either higher or lower doses. This primary increase in mitochondrial ROS is also shown to have effects on mitochondrial function, [Ca^2+^]_I_ homeostasis and cell viability in a dose-dependent manner.

Until this point, studies assessing the effects of the alteration of intracellular ROS levels have necessarily used crude approaches to modulate ROS, which poorly mimic the (patho)physiological generation of ROS. For example, the application of exogenous hydrogen peroxide or exposure to a purine/xanthine oxygen radical generating system both require the ROS to diffuse into the cell, rather than being generated selectively within cell compartments such as the mitochondria. These limitations have been addressed by using MitoPQ [31]. MitoPQ's mitochondrial accumulation allows it to be used at concentrations several hundred-fold lower than untargeted paraquat and importantly this selective accumulation means that ROS are only produced within mitochondria. The direct generation of superoxide rather than downstream ROS also mirrors its production in (patho)physiology as the proximal species [53]. This has been validated to occur without detectable ROS production in the cytosol [54]. The dose-dependent effect of MitoPQ on mitochondrial ROS production therefore allows the generation of exogenous ROS in a highly specific manner. Based on these features, MitoPQ represents a unique tool to investigate and characterize the effects of a precise increase in mitochondrial ROS formation on cell physiology, both *in vitro* and *in vivo*.

There is a growing body of evidence indicating that mitochondrial ROS exert differential effects depending on their dose, which has been called a “hormetic” dose-response curve [55]. In our experiments, high levels of ROS induced by MitoPQ (i.e. 0.5–1 μM) lead to mitochondrial dysfunction, mPTP opening and eventually cell death. Such detrimental effects of ROS in I/R injury through the opening of the mPTP have been well described [5]. Intermediate doses of ROS (i.e. 0.1 μM MitoPQ) did not affect cell viability but did cause an alteration in the amplitude of calcium transients and their response to caffeine. At low doses (i.e. 0.01 μM), ROS generation induced by MitoPQ slightly modulates [Ca^2+^]_I_ homeostasis without affecting either mitochondrial function or cell viability. Importantly, isolated cardiomyocytes pre-treated with low doses of MitoPQ and then exposed to anoxic injury displayed increased cell viability. This *in vitro* result was paralleled by the reduction of infarct size obtained *in vivo* in mice pre-treated with doses of MitoPQ ranging from 0.01 nmol to 1 nmol per mouse. Therefore, mitochondrial-derived ROS elicit a wide range of responses which are dependent on their dose, ranging from the disruption of ΔΨm and mPTP opening at high doses of MitoPQ to the reduction of cell death and infarct size at low doses of MitoPQ in a preconditioning-like manner. The absence of protective effects observed with both low and high doses of MitoPQ *in vivo* (i.e. 0.001–1 nmol) highlights the hormetic effect elicited by intermediate levels of ROS. Moreover, higher doses of MitoPQ (i.e. 2.5 – 10 nmol) displayed to be lethal *in vivo*. The concept of hormesis helps to rationalize the paradoxical effect of both injury and protection elicited by ROS described in literature, as well as the failure of clinical trials using general administration of antioxidants.

The data presented also show that a number of ROS-related events spread from mitochondria to the cytosolic compartment. As the source of ROS generation in our experiments is within mitochondria, we provide the first direct evidence that a primary increase in mitochondrial ROS formation can induce cardioprotection both *in vitro* and *in vivo* via changes in mitochondrial and cellular function. The known interplay between ROS and [Ca^2+^]_I_ homeostasis is of central relevance to the cardiomyocyte and has specifically been implicated in several disease states [56], but it has been difficult to determine the primacy of one factor upon the other. Taken together, our findings show that a primary increase in mitochondrial ROS can impact on the cytosol, altering [Ca^2+^]_I_ homeostasis and thereby whole cell function.

It is worth noting that while we have determined the doses of MitoPQ associated with the various effects in mitochondria and in intact cells, the actual concentrations of ROS required for cardiomyocytes injury and protection remain to be established due to the technically challenging nature of their measurement, especially *in vivo* [57]. Moreover, since we have utilised healthy young male animals for our *in vivo* model it remains to be seen how comorbidities such as obesity, diabetes, aging and sex modulate the response to a given quantity of mitochondrial ROS, since for example the protective efficacy of IPC is lost in aged or diabetic hearts [51].

In conclusion, we have demonstrated that a primary increase in mitochondrial ROS exerts effects on both mitochondrial and cell function, which presents mitochondrial ROS as a cause rather than consequence of the functional change in cardiac (patho)physiology. Notably, the generation of exogenous ROS by MitoPQ within mitochondria is found to exert differential effects, with a hormetic dose response curve in which protection is observed only at an intermediate level of mitochondrial ROS, but not at either lower or higher levels.

## 4 Methods

### 4.1 Cell culture

#### 4.1.1 NRVMs

Neonatal rat ventricular myocytes (NRVMs) were isolated from 1 to 3 day old Wistar rats as described previously [58]. Briefly, hearts were excised, cut into smaller pieces and left overnight at 4 °C for digestion by 2.5% trypsin 10× (Thermo Fisher Scientific) in HBSS (Sigma). The next day, tissues were incubated with 0.75 mg/ml collagenase type II (Thermo Fisher Scientific) in HBSS for 10 min (at 2 min intervals) at 37 °C and cells dissociated by pipetting. Following centrifugation at 300*g* for 7 min, cells were resuspended in MEM (Invitrogen) and pre-plated for 2 h to let cardiac fibroblasts attach to the plastic surface. Plates and coverslips were coated with a solution of 0.1% porcine gelatin (Sigma) and incubated at 37 °C for 1 h. The non-adherent myocytes were plated in gelatin coated plates at variable density (at least 1 × 10^5^ cells/ml) in MEM supplemented with 10% FBS (Thermo Fisher Scientific), 1% penicillin/streptomycin (Thermo Fisher Scientific), 1% non-essential amino acids (Thermo Fisher Scientific), 1 mM 5-Bromo-2-Deoxyuridine (Sigma). Cells were maintained at 37 °C in presence of 5% CO_2_. The medium was changed to MEM supplemented with 1% FBS, 1% penicillin/streptomycin and 1% non-essential amino acids after 24 h of plating.

To evoke a primary increase in mitochondrial ROS, NRVMs were treated in culture medium with different concentrations of MitoPQ [31], from 0.01 to 1 μM for 2 h, unless specified in results. A MitoPQ control compound was also used that has a very similar structure to MitoPQ and similar levels of uptake into mitochondria, but which cannot generate ROS by redox cycling. MitoPQ redox cycles because it can receive an electron from the FMNH_2_ of complex I reducing the viologen dication to a radical cation, which then reduces oxygen to superoxide to regenerate MitoPQ (Supplemental Fig. 1). MitoPQ control employs the twisted viologen unit described by Ref. [59], in which two extra methyl groups disfavour the coplanarity of the two pyridine units required to stabilize a radical cation. We have previously reported its use [60], but here give full details of its synthesis in two steps by double alkylation of 3’-dimethyl-4,4’-dipyridyl, prepared by the procedure of (Rebek et al., 1985) (Supplementary Fig. 3). To scavenge ROS, cells were pre-treated with 500 μM MPG (Sigma) [61] for 30 min. To prevent mPTP opening, cells were pre-treated with 1 μM CsA (Sigma) [62] for 30 min.

#### 4.1.2 Transfection

NRVMs were plated on six-well plates at a density of 3 × 10^5^ cells/well and transfected with Lipofectamine 3000 reagent (Sigma). For each transfection, 2.5 μg of MitoHyPer (Evrogen) was diluted in 125 μl of Opti-MEM medium (Thermo Fisher Scientific) in presence of 5 μl of P3000™ reagent (Life Technologies) and later combined with 4 μl of Lipofectamine™ 3000 (Life Technologies). The DNA-lipid complexes were added to the cells and incubated overnight. The day after, cells were rinsed with PBS and new MEM was added. Transfected cells were used for experiments after 48 h.

#### 4.1.3 Imaging

Experiments using NRVMs were carried out in HBSS at pH 7.4 (adjusted with NaOH) and at 37 °C.

Images were acquired using an inverted fluorescence microscope (Leica DMI6000B equipped with DFC365FX camera) with PL APO 40×/1.25 oil objective. Fluorescence intensity was quantified using the Fiji distribution of the Java-based image processing program ImageJ [63], and background signal was subtracted from all analysed regions of interest. For Ca^2+^ imaging, traces were analysed using the “Peak Analyzer” tool of Origin Pro 9.1.

To monitor mitochondrial ROS formation, cells were incubated with 25 nM MitoTracker Red CM-H_2_XRos (MTR, Thermo Fisher Scientific) for 30 min at 37 °C in a humidified incubator. Since the accumulation of MTR in NRVMs can vary from different preparation, data were normalized to DMSO control.

To monitor mitochondrial membrane potential (ΔΨm), cells were incubated with 25 nM tetramethylrhodamine (TMRM, Thermo Fisher Scientific) in presence of 1.6 μM cyclosporin H (CsH) for 30 min at 37 °C in a humidified incubator. TMRM fluorescence intensity was monitored following addition of 4 μM oligomycin (Sigma) and images were acquired before and after the addition of 4 μM carbonyl cyanide-p-trifluoromethoxyphenylhydrazone (FCCP, Sigma) [58]. In order to have a reliable value of TMRM, fluorescence values were expressed as ΔF (F_0_/F_FCCP_) and results were normalized to DMSO control basal value.

To monitor mPTP opening, cells were incubated with 1 μM calcein acetoxymethyl (AM) ester (Thermo Fisher Scientific) in presence of 1 mM Cobalt Chloride (CoCl_2_) for 15 min at 37 °C in a humidified incubator as previously described [42]. To evaluate the extent of pore opening, data were normalized to the basal value.

To monitor [Ca^2+^]_I_ homeostasis, cells were incubated with 5 μM Fluo-4 AM ester (Thermo Fisher Scientific), 0.01% w/v pluronic F-127 (Sigma) and 250 μM sulfinpyrazone (Sigma), for 20 min at 37 °C in MEM followed by 20 min of de-esterification. Since the accumulation of Fluo-4 in NRVMs can vary from different preparation, data were normalized to DMSO control.

#### 4.1.4 Assessment of cell death

For normoxic experiments, NRVMs were seeded in 24w plates at density of 10^5^ cells/well and cultured in MEM supplemented with 1% FBS, 1% penicillin/streptomycin and 1% non-essential amino acids. Cells were incubated with different concentrations of MitoPQ with or without MPG or CsA for 24 h at 37 °C in a humidified incubator.

For anoxia/reperfusion experiments, NRVMs were seeded in 24w plates at density of 10^5^ cells/well and incubated in 118 mM NaCl, 5 mM KCl, 1.2 mM KH_2_PO_4_, 1.2 mM MgSO_4_, 2 mM CaCl_2_, 25 mM MOPS at pH 6.4 during anoxia or pH 7.4 during reoxygenation [64]. Anoxia was induced adding 10 mM 2-deoxy-d-glucose (2-DG) and incubating in a BD GasPak™ EZ Anaerobe Gas-generating Pouch System with an indicator (BD Biosciences) at 37 °C for 12 h [65]. To induce reoxygenation, plates were removed from the GasPak™ pouch, 2-DG was replaced with 10 mM d-glucose, the pH was restored at 7.4. The plates were then incubated for 1 h in a humidified incubator at 37 °C.

The release of LDH from NRVMs was measured to evaluate cell death occurring in normoxia, anoxia and reoxygenation as described before [62,66]. Supernatant aliquots were collected after 24 h of normoxia, 12 h of anoxia and 1 h of reoxygenation. At the end of every experiment, intact cells were lysed by incubating with 1% Triton X-100 (Sigma) for 30 min and supernatants were collected to evaluate the total amount of LDH. LDH enzymatic activity was measured spectrophotometrically by the absorbance of nicotinamide adenine dinucleotide (Roche) at 340 nm, indicative of the reduction of pyruvate to lactate.

### 4.2 Experimental animals

Male C57BL/6 J mice aged 8–10 weeks (22–32 g) were obtained from Charles River, UK. They were housed under standard laboratory conditions, with food and water available *ad libitum*. All procedures were carried out in accordance with the UK Home Office Guide on the Operation of Animal (Scientific Procedures) Act 1986 and University of Cambridge Animal Welfare Policy under project licenses 70/8238 and 70/7963.

### 4.3 Open-chest mouse model of acute myocardial I/R injury

An open chest model of acute myocardial ischaemia/reperfusion injury was used as described elsewhere [67]. Mice were anesthetized with sodium pentobarbital (70 mg/kg intraperitoneal), with depth of anaesthesia monitored via the pedal reflex and additional anaesthesia administered as required. Following left side lateral thoracotomy, the left anterior descending coronary artery was occluded for 30 min followed by 2 h of reperfusion. Compounds were administered 15 min prior to the start of ischaemia by an intravenous bolus injection in the lateral tail vein.

At the end of the protocol, the area at risk was delineated by retrograde injection of 10 mg/ml Evans Blue after re-occlusion of the left anterior descending coronary artery. Heart sections were incubated for 25 min at 37 °C in 1% triphenyltetrazolium chloride (Sigma, UK) before fixing for 24 h in 10% formalin. Planimetry was performed in a blinded fashion using ImageJ [68]. Hearts in which the area at risk was outside of the range 30%–60% of total area were excluded from any further analysis.

### 4.4 Pressure-volume analysis of cardiac function

Anaesthesia was induced with 3% isoflurane in O_2_ in a plexiglass chamber. Mice were transferred to a heated surgical platform, and sufficient isoflurane administered to maintain anaesthesia as assessed by the pedal reflex. Body temperature was maintained at 37 °C using a rectal thermometer and temperature controller (TCAT-2LV, Physitemp, USA). The left ventricle was catheterized via the right carotid artery with a 1.2 French tetrapolar catheter (Transonic Scisense Inc, Canada) as described elsewhere [69]. In brief, a small midline incision was made in the neck in order to expose the carotid artery and isolate it from the vagus nerve. A 4-0 silk suture was tied tightly around the distal end of the artery, and two more sutures were placed loosely at the proximal end. Using a vascular clamp (0.4–1 mm) to minimise blood loss, a small incision was made in the carotid artery with microscissors and the catheter inserted and secured using the additional sutures. The catheter was the inserted along the carotid until it was located centrally within the left ventricle, as indicated by the phase signal and by the shape of the resultant pressure-magnitude loops. At least 15 min were allowed for the haemodynamics to stabilize. A 100 μL bolus containing either 0.1 nmol MitoPQ or vehicle only was then injected via the lateral tail vein. Data were recorded at 1000 Hz using the ADV500 PV system (Transonic Scisense Inc, Canada) and a multi-channel acquisition system (Powerlab, ADInstruments, UK) and were analysed in LabChart (ADInstruments, UK). Volumes were calculated on dynamic basis using Wei's equation [70]. Three short sections of loops (within one breathing cycle) were examined at both baseline conditions and >5 min following injection. Upon completion of the protocol all animals were killed via cervical dislocation.

### 4.5 Chemical synthesis

MitoPQ was synthesized from iododecyl-TPP salt **1**, which was prepared as described previously [31] (Supplementary Fig. 3). This was reacted with an excess of 3,3'-dimethyl-4,4’-dipyridyl **2**, prepared by the procedure of (Rebek et al. [71]), to minimise dialkylation. The monoalkylated product **3** was isolated in excellent yield and was then methylated to give complete conversion to MitoPQ control. Original NMR spectra for MitoPQ control and compound 3 can be found at 10.5525/gla.researchdata.735.

#### 4.5.1 3-Methyl-4-(3“-methylpyrid-4“-yl)-1-(10‴-triphenylphosphoniodec-1‴-yl)pyridinium diiodide 3

(10-Iododec-1-yl)triphenylphosphonium iodide **1** (107 mg, 0.163 mmol, 1.0 eq.) was added to a solution of 3,3’-Dimethyl-4,4’-dipyridyl **2** (120 mg, 0.65 mmol, 4.0 eq) in MeCN (2 ml) and the resulting solution was heated to 60 °C overnight under an atmosphere of argon. The solution was cooled to RT and concentrated under vacuum. Column chromatography eluting with CH_2_Cl_2_-MeOH (100:0 to 85:15) then gave the pyridinium salt **3** as an off-white solid (116 mg, 85%). ν_max_ (ATR): 2926 (CH), 2854 (CH), 1635 (Ar), 1437 (CH) cm^−1^. *δ*_H_ (400 MHz, CDCl_3_): 9.98 (1H, s, H-1), 9.71 (1H, d, *J* = 6.3 Hz, H-2), 8.62 (1H, s, H-4), 8.58 (1H, d, *J* = 5.0 Hz, H-5), 7.86–7.68 (16H, m, PPh_3_ + H-3), 7.06 (1H, d, *J* = 4.9 Hz, H-6), 4.97 (2H, t, *J* = 7.8 Hz, NCH_2_), 3.61–3.51 (2H, m, PCH_2_), 2.37 (3H, s, CH_3_), 2.32–2.16 (2H, m, NCH_2_*CH*_*2*_), 2.10 (3H, s, CH_3_), 1.70–1.60 (8H, m, 4 × CH_2_), 1.59–1.50 (2H, m, CH_2_), 1.49–1.25 (4H, m, 2 × CH_2_). *δ*_C_ (101 MHz, CDCl_3_): 154.85 (C), 151.42 (CH), 147.46 (CH), 145.16 (CH), 142.50 (CH), 137.03 (C), 134.94 (d, *J* = 3.0 Hz, CH), 133.25 (d, *J* = 10.0 Hz, CH), 130.32 (d, *J* = 12.6 Hz, CH), 129.32 (C), 127.45 (CH), 121.39 (CH), 117.60 (d, *J* = 86.0 Hz, C), 60.75 (CH_2_), 31.17 (CH_2_), 29.81 (d, *J* = 15.8 Hz, CH_2_), 28.26 (CH_2_), 28.17 (CH_2_), 27.99 (CH_2_), 27.96 (CH_2_), 25.41 (CH_2_), 22.70 (d, *J* = 50.4 Hz, CH_2_), 22.11 (d, *J* = 4.4 Hz, CH_2_), 16.81 (CH_3_), 16.48 (CH_3_). δ_P_ (162 MHz: CDCl_3_): 23.94 (s). m/z (ESI): Found: 293.1734. C_40_H_47_N_2_P requires (*M*^*2+*^), 293.1733.

#### 4.5.2 1,3,3’-trimethyl-1’-(10‴-triphenylphosphoniodec-1‴-yl)-4,4’-bipyridinium (MitoPQ control) triiodide

Iodomethane (19 μl, 0.29 mmol, 5.0 eq.) was added to a solution of pyridine **3** (49 mg, 0.058 mmol, 1.0 eq) in MeCN (1 ml) and the resulting solution was heated to 40 °C overnight under an atmosphere of argon. The solution was cooled to RT and concentrated under vacuum to give the MitoPQ triiodide as an off-white solid (57 mg, 100%). ν_max_ (ATR): 3016 (CH), 2926 (CH), 2854 (CH), 1635 (Ar), 1437 (CH) cm^−1^. *δ*_H_ (400 MHz, d3-MeCN): 9.14 (1H, s, H-1), 8.94 (1H, s, H-4), 8.91 (1H, d, *J* = 6.3 Hz, H-2), 8.77 (1H, d, *J* = 6.3 Hz, H-5), 7.99–7.86 (5H, m, H-3, H-6 + 3 × ArH), 7.81–7.71 (12H, m, 12 × ArH), 4.67 (2H, t, *J* = 7.6 Hz, NCH_2_), 4.42 (3H, s, NCH_3_), 3.35–3.23 (2H, m, PCH_2_), 2.30 (3H, s, CH_3_), 2.29 (3H, s, CH_3_), 2.07 (2H, q, *J* = 7.3 Hz, NCH_2_*CH*_*2*_), 1.70–1.60 (2H, m, PCH_2_*CH*_*2*_), 1.58–1.49 (2H, m, CH_2_), 1.47–1.25 (10H, m, 5 × CH_2_). *δ*_C_ (101 MHz, d3-MeCN): 152.15 (C), 147.69 (CH), 146.81 (CH), 143.92 (CH), 143.12 (CH), 138.41 (C), 137.98 (C), 135.86 (d, *J* = 3.0 Hz, CH), 134.55 (d, *J* = 10.0 Hz, CH), 130.07 (d, *J* = 12.5 Hz, CH), 128.04 (CH), 127.81 (CH), 119.31 (d, *J* = 86.2 Hz, C), 62.20 (CH_2_), 49.19 (CH_3_), 31.74 (CH_2_), 30.76 (d, *J* = 16.2 Hz, CH_2_), 29.55 (CH_2_), 29.48 (CH_2_), 29.30 (CH_2_), 29.04 (CH_2_), 26.35 (CH_2_), 22.81 (d, *J* = 4.4 Hz, CH_2_), 22.61 (d, *J* = 50.7 Hz, CH_2_), 17.54 (CH_3_), 17.41 (CH_3_). δ_P_ (162 MHz: d3-MeCN): 23.84 (s). m/z (ESI): Found: 200.4568. C_41_H_50_N_2_P requires (*M*^*3+*^), 200.4565.

### 4.6 Data analysis

All values are expressed as mean ± S.E.M. Every set of data has 3 biological replicates (i.e. 3 different cardiac preparations) and every biological replicate has at least 3 technical replicates (i.e. 3 different samples of the same preparation). The propagation of error analysis was performed to take into account the uncertainty that is present in the experimental measurements due to measurement limitations (i.e. different accumulation of the sensor in NRVMs derived from different preparations). Comparison between groups was performed by one-way ANOVA, followed by post hoc testing (i.e. Tukey's range test, Dunnett's test) adjusted for multiple comparisons where data were normally distributed. Data that did not follow the normal distribution were statistically analysed by Kolgomorov-Smirnov's test. Comparison between two groups was performed using a two-tailed Student's t-test, with correction for multiple testing by the Bonferroni method where appropriate. A value of *p* < 0.05 was considered significant.

## Abbreviations

[Ca^2+^]_I_: intracellular calcium concentration
A/R: anoxia/reoxygenation
CsA: cyclosporine A
IPC: ischaemic preconditioning
I/R: ischaemia/reperfusion
LDH: lactate dehydrogenase
MitoPQ: MitoParaquat
MPG: N-(2-Mercaptopropionyl)glycine
mPTP: Mitochondrial permeability transition pore
MTR: mitotracker red CMH_2_X-ROS
NRVM: neonatal rat ventricular myocytes
TMRM: tetramethylrhodamine
ΔΨm: mitochondrial membrane potential
