# Supplementary material for: Selective mitochondrial superoxide generation in vivo is cardioprotective through hormesis
Source: Free Radic Biol Med. 2019 Apr;134:678–87. doi: 10.1016/j.freeradbiomed.2019.01.034 (PMC6607027; doi:10.1016/j.freeradbiomed.2019.01.034)
Supplement: Multimedia component 1 [file mmc1.pdf]

SUPPLEMENTARY TABLE AND FIGURES

	Vehicle		MitoParaquat	
	mean fold change	SEM	mean fold change	SEM
Cardiac Output	1.17	0.15	1.13	0.22
Heart rate	1.00	0.00	0.98	0.00
Ejection fraction	1.00	0.04	0.99	0.05
Stroke Volume	1.17	0.15	1.15	0.23
End systolic volume	1.15	0.17	1.23	0.16
End diastolic volume	1.10	0.13	1.12	0.18
End systolic pressure	1.01	0.03	0.98	0.02
End diastolic pressure	1.32	0.34	1.30	0.28
Stroke Work	1.13	0.14	1.06	0.23
Mean pressure	1.05	0.05	1.02	0.04
Developed pressure	0.99	0.00	0.97	0.01
dP/dt_max	0.91	0.05	0.86	0.03
dP/dt_min	0.98	0.05	0.88	0.03
dV/dt_max	1.27	0.30	1.24	0.25
dV/dt_min	1.34	0.27	1.10	0.25
Pressure at dV/dt_max	1.03	0.23	1.08	0.28
Pressure at dP/dt_max	0.97	0.01	0.93	0.01
Volume at dP/dt_max	1.17	0.12	1.15	0.21
Volume at dP/dt_min	1.19	0.18	1.24	0.16

Table S1. MitoPQ does not affect haemodynamics. A closed chest experimental model was used to assess the effect of MitoPQ on haemodynamics. A pressure-volume catheter using admittance technology was inserted into the left ventricle via the right carotid artery. No significant difference in the fold change of values of any of the parameters measured following i.v. injection, compared between MitoPQ (0.1 nmol) or vehicle only. n = 4, Student's t-test with Bonferroni correction for multiple comparisons.

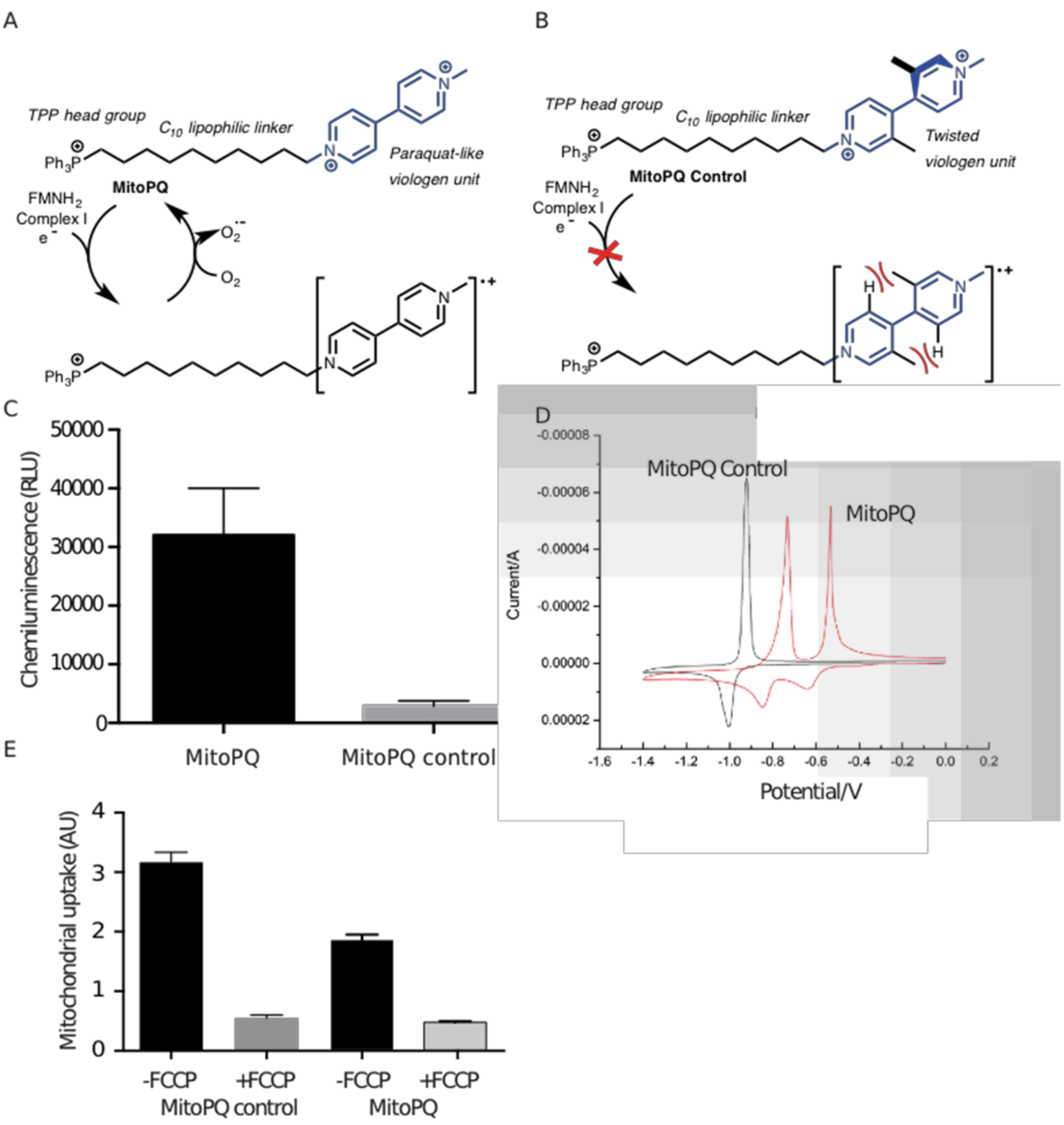

<u>Supplementary Figure 1. Comparison of MitoPQ and MitoPQ Control.</u> A) MitoPQ is comprised of an alkyltriphenylphosphonium (TPP) head group for targeting to the mitochondrial matrix, a lipophilic alkyl linker and a viologen head group similar to that of paraquat (spectator counterions omitted for clarity). The viologen is reduced by $FMNH_2$ of complex I to give the corresponding radical cation, in which the charge and radical character is delocalized over the two pyridine rings. Rapid reaction with molecular oxygen then produces superoxide and regenerates MitoPQ. **B)** The two methyl groups of MitoPQ control induce a twist in the viologen unit and the compound is less easily reduced because the coplanarity required for a delocalized radical cation is disfavoured. Thus, complex I is not sufficiently reducing to reduce MitoPQ control. **C)** *Superoxide production.* Bovine heart mitochondrial membranes (70 µg protein) were incubated in KCl buffer (120 mM KCl, 10 mM Hepes, 1 mM EGTA, pH 7.4) with 4 µg/ml rotenone, 2 µM coelentrazine and 1 mM NADH ± 1 µM MitoPQ or MitoPQ control in a 1 ml final volume. Coelentrazine luminescence was recorded on a Berthold Autolumat Plus luminometer at 30°C. **D)** *Cyclic voltammograms of MitoPQ and MitoPQ control (1x10^{-3} M in pH 7.4 phosphate buffer)*. The reference electrode was Ag/AgCl and was measured at scan rate of 0.05 V/s. The working electrode was a glassy carbon disk and the counter electrode was platinum wire. As expected from the literature (Andersson et al., 2009), while MitoPQ has a first reduction half potential of –0.59 V, MitoPQ control is much more difficult to reduce with a reduction half potential of –0.97 V in pH 7.4 phosphate buffer. **E)** *Uptake by isolated mitochondria.* Rat liver mitochondria (1 mg/ml) were incubated in KCl buffer (120 mM KCl, 10 mM Hepes, 1 mM EGTA) with succinate (10 mM), rotenone (4 µg/ml), TPMP (5 µM), and MitoPQ or MitoPQ control (5 µM) ± FCCP (0.5 µM) at 37°C for 5 min. The mitochondria were then pelleted by centrifugation (5 min at 10000 x g). The pellet was disrupted by vortexing with 250 µl of 99.9% ACN + 0.1% TFA and centrifuged again (5 min at 10000 x g). The supernatant was removed and added to 750 µl 99.9% water + 1% TFA and analysed by RP-HPLC with peak areas recorded.

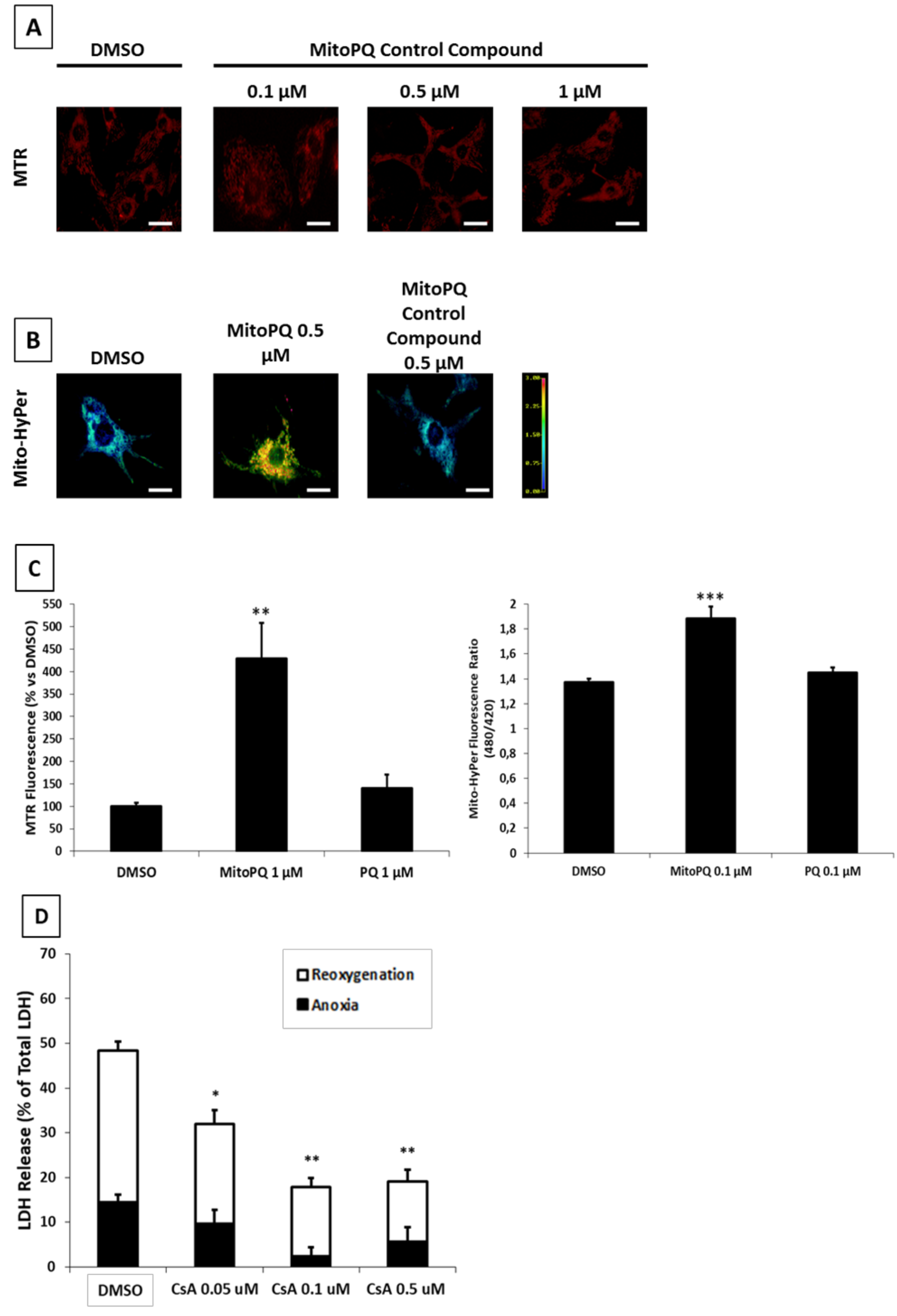

Supplementary Figure 2. Effect of MitoPQ Control Compound and PQ on ROS formation and effect of CsA in NRVMs exposed to A/R injury. A) Mitochondrial ROS formation monitored by MTR in isolated NRVMs treated for 2 h with different concentrations of MitoPQ Control Compound (0.1 – 0.5 – 1 µM). *Representative images. Scale Bar: 30 µm* **B)** Mitochondrial H_2O_2 formation measured by MitoHyPer in isolated NRVMs treated for 2 h with 0.5 µM MitoPQ or 0.5 µM MitoPQ Control Compound. *Representative images. Scale Bar: 20 µm* **C)** Mitochondrial ROS and H_2O_2 formation measured by MTR and MitoHyPer in isolated NRVMs treated for 2 h with different concentrations of MitoPQ (0.1 – 1 µM) or PQ (0.1 – 1 µM). **D)** Cell death measured by LDH release from isolated NRVMs exposed to 12 hours of anoxia and 1 hour of reoxygenation in presence or absence of different doses of CsA (0.05 – 0.1 – 0.5 µM). $^*p < 0.05$, $^{**}p < 0.01$ vs DMSO Reoxygenation.

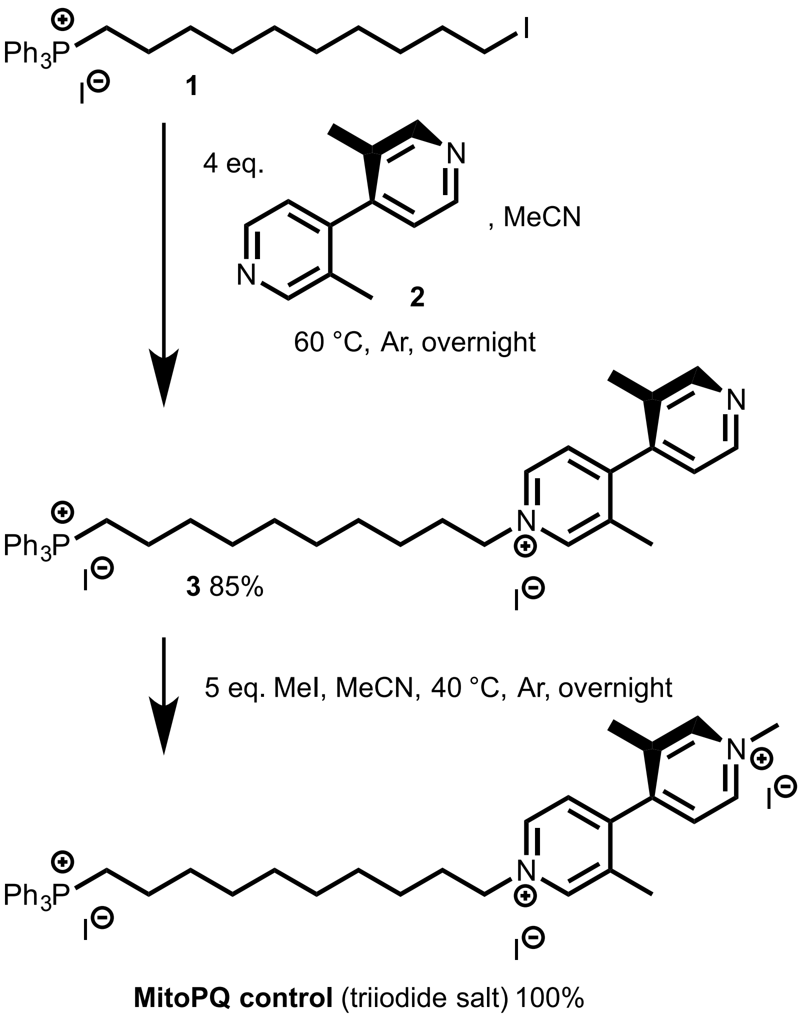

Supplementary Figure 3. Synthesis of MitoPQ control. MitoPQ was synthesized from iododecyl-TPP salt 1, which was prepared as described previously (Robb et al.). This was reacted with an excess of dimethyl-4,4'-dipyridyl 2, prepared by the procedure of (Rebek et al.), to minimise dialkylation. The monoalkylated product 3 was isolated in excellent year and was then methylated to give complete conversion to MitoPQ control.
